# Role of Inositol Phosphosphingolipid Phospholipase C1, the Yeast Homolog of Neutral Sphingomyelinases in DNA Damage Response and Diseases

**DOI:** 10.1155/2015/161392

**Published:** 2015-08-06

**Authors:** Kaushlendra Tripathi

**Affiliations:** Department of Oncologic Sciences, Mitchell Cancer Institute, University of South Alabama, 1660 Springhill Avenue, Mobile, AL 36604, USA

## Abstract

Sphingolipids play a very crucial role in many diseases and are well-known as signaling mediators in many pathways. Sphingolipids are produced during the *de novo* process in the ER (endoplasmic reticulum) from the nonsphingolipid precursor and comprise both structural and bioactive lipids. Ceramide is the central core of the sphingolipid pathway, and its production has been observed following various treatments that can induce several different cellular effects including growth arrest, DNA damage, apoptosis, differentiation, and senescence. Ceramides are generally produced through the sphingomyelin hydrolysis and catalyzed by the enzyme sphingomyelinase (SMase) in mammals. Presently, there are many known SMases and they are categorized into three groups acid SMases (aSMases), alkaline SMases (alk-SMASES), and neutral SMases (nSMases). The yeast homolog of mammalians neutral SMases is inositol phosphosphingolipid phospholipase C. Yeasts generally have inositol phosphosphingolipids instead of sphingomyelin, which may act as a homolog of mammalian sphingomyelin. In this review, we shall explain the structure and function of inositol phosphosphingolipid phospholipase C1, its localization inside the cells, mechanisms, and its roles in various cell responses during replication stresses and diseases. This review will also give a new basis for our understanding for the mechanisms and nature of the inositol phosphosphingolipid phospholipase C1/nSMase.

## 1. Introduction

In the eukaryotic cell, sphingolipids make up an imperative set of regulatory molecules and they are involved in a broad array of cellular activities such as cell growth, inflammation, checkpoint, angiogenesis, and stress stimuli [1–3]. Baker's yeast* Saccharomyces cerevisiae* has been used by various researchers as a model for studying the sphingolipid biosynthesis because of its ease in sphingolipid metabolism as compared to mammalian systems [3, 4]. The set of bioactive sphingolipids includes ceramide, sphingosine, sphingosine-1-phosphate, and ceramide-1-phosphate. The mammalian neutral sphingomyelinase (nSMase 2) ortholog in* yeast* is inositol phosphosphingolipid phospholipase C1 (*ISC1*). Bacterial nSMase sequence analysis facilitated in the revealing of yeast nSMase inositol phosphosphingolipid phospholipase C1 and nSMases of mammals. It is encoded by the inositol phosphosphingolipid phospholipase C gene in* Saccharomyces cerevisiae* and* Cryptococcus neoformans* [5, 6]. In fission yeast* Schizosaccharomyces pombe*, it is encoded by the gene* CSS1* known as Can't Stop Synthesizing cell wall and* ISCL* gene in* Leishmania major. ISC1*/nSMase 2 are well-known to catalyze the degradation of complex phosphosphingolipids into phytoceramide/dihydroceramide in* Saccharomyces cerevisiae* and ceramide/dihydroceramide in mammals [7]. Ceramides are well studied bioactive lipids and known to play a crucial role in various cell functions such as senescence, growth, and apoptosis and at the time of stress [8]. Yeast cells lacking inositol phosphosphingolipid phospholipase C exhibit a slower growth rate than its wild type strains [9]. These cells also show higher sensitivity to high salt concentration (NaCl), hydrogen peroxide and and lead to G2/M arrest after hydroxyurea drug treatment [5, 10]. Various groups have also shown that inositol phosphosphingolipid phospholipase C protein plays an important role not only in chronological lifespan but also in oxidative stress resistance [10, 11]. Inositol phosphosphingolipid phospholipase C also regulates the cellular redox homeostasis via inflection of iron levels. Loss of inositol phosphosphingolipid phospholipase C gene decreased cells hypersensitive to hydrogen peroxide, hydroxyurea, and methyl methanesulfonate compared with the wild type cells. A genome-wide transcriptome analysis revealed that the deletion of inositol phosphosphingolipid phospholipase C leads to an increase of messenger RNA levels of nearly about seventy-two genes and a decline of nearly about 142 genes. This clearly indicates that inositol phosphosphingolipid phospholipase C plays a crucial role in the cells.

In pathogenic fungi,* Cryptococcus neoformans* inositol phosphosphingolipid phospholipase C is also known to metabolize the fungal inositol sphingolipids [12]. It is also well-known that inositol phosphosphingolipid phospholipase C enhances* Cryptococcus neoformans* endurance in macrophages that is vital for controlling the spreading of this infectious pathogen into the brain [13]. The inositol phosphosphingolipid phospholipase C deleted (*isc1Δ*) mutant of* Cryptococcus neoformans* strain shows another characteristic feature of hyperencapsulation. Inositol phosphosphingolipid phospholipase C, fission yeast homolog Css1p hydrolyzed IPC to the same level as inositol phosphosphingolipid phospholipase C in baker's yeast. Furthermore, the genome-wide screen also revealed that inositol phosphosphingolipid phospholipase C deleted (*isc1Δ*) mutants were sensitive to methyl methanesulfonate (MMS), a DNA alkylating agent and the DNA replication blocking drug hydroxyurea [14]. In the later section of this review, we will discuss more about the effects of these drugs.

## 2. Sphingolipids in Yeast

Yesteryear, the baker's yeast* Saccharomyces cerevisiae* has emerged as a great tool in the study of sphingolipid function and its metabolism. Firstly, in yeast most of the key enzymes involved in the metabolism of bioactive sphingolipids were identified. Stress stimuli such as heat stress play a crucial role in the regulation of acute production of bioactive sphingolipids in yeast [1, 15]. Sphingolipids have been involved in mediating several important responses into the cell such as cell cycle arrest and regulation of protein translation. One of the important steps that appears to act in sphingolipid mediated signal pathway is the activation of the inositol phosphosphingolipid phospholipase C. Inositol phosphosphingolipid phospholipase C has substantial similarity to mammalian SMases that have direct effect on animal sphingolipid signaling pathway [5]. In mammals, at present there are five different kinds of nSMases known, such as nSMase 1, nSMase 2, nSMase 3, and mitochondrial associated nSMase (MA-nSMase), which are decoded by five genes* SMPD2* (sphingomyelin phosphodiesterase 2),* SMPD3*,* SMPD4*, and* SMPD5,* respectively.

**Figure 1:**
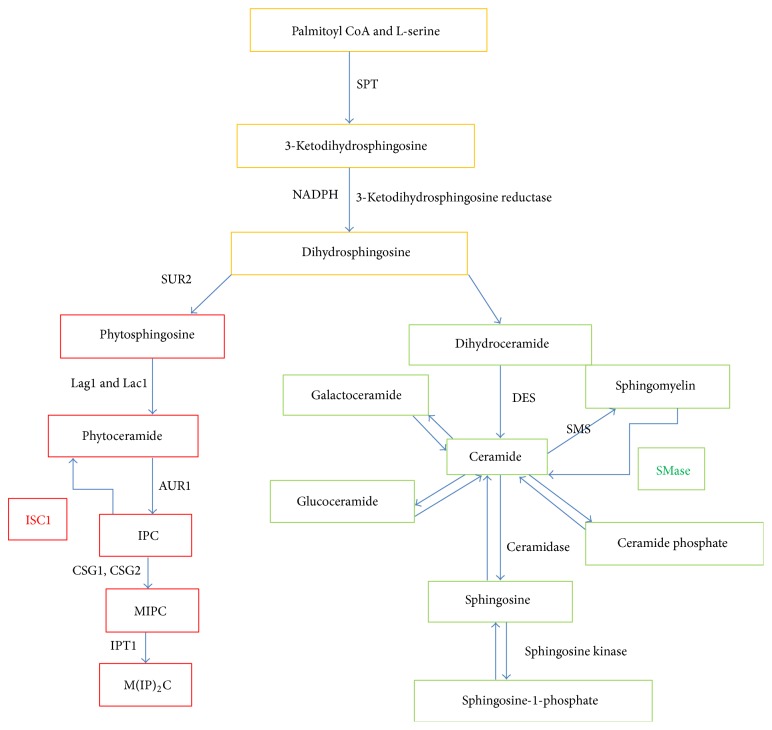
The flow diagram represents the sphingolipid metabolism pathways both in mammals and in yeast. The common steps in both the organisms are shown in yellow boxes. The yeast sphingolipid metabolism pathway steps are shown in red boxes while mammals pathway are shown in green boxes; the positions of sphingomyelinase (SMase) and inositol phosphosphingolipid phospholipase C1 (*ISC1*) are shown in green and red letters, respectively.

The sphingolipid biosynthesis starts with the condensation process of fatty acyl-CoA and serine through the enzyme complex serine palmitoyltransferase (SPT) (Figure 1) [16]. Serine palmitoyltransferase complex enzyme consists of two major subunits which are coded by two different genes long chain base 1 (*LCB1/YMR296C*) and long chain base 2 (*LCB2/YDR062W)* and a third small subunit, encoded by the gene* TSC3* (temperature-sensitive suppressor of* csg2Δ*). In mammals, serine palmitoyltransferase complex enzyme is encoded by three different genes* SPTLC1* (mutation in it linked to sensory neuropathy, type 1),* SPTLC2*, and* SPTLC3*. It requires pyridoxal-5′-phosphate as a cofactor and endures functional and sequence homology to *α*-oxoamine synthases group of enzymes [17, 18].* In vivo* and* in vitro* studies unambiguously suggest that both serine palmitoyltransferase 1 (Lcb1p) and serine palmitoyltransferase 2 (Lcb2p) are required for serine palmitoyltransferase complex activity. Though, point mutants in* LCB2* amplified serine palmitoyltransferase complex activity in the absence of* TSC3*. This indicates that Tsc3p mediates serine palmitoyltransferase activation probably through interaction with the subunit of Lcb2p [19, 20]. The serine palmitoyltransferase complex produces 3-ketodihydrosphingosine that is quickly converted into dihydrosphingosine in an NADPH dependent way by the enzyme 3-ketodihydrosphingosine reductase decoded by the gene* TSC10/YBR265w* (temperature-sensitive suppressor of* csg2Δ*) [21]. In mouse and human, it is encoded by the genes known as the human FVT-1 (hFVT-1) and mouse FVT-1 (mFVT-1) [22]. These steps clearly suggest that yeast and mammals share a lot of similarities at the initial steps of sphingolipid metabolism pathway [23].

Differences in the sphingolipid metabolism pathways between yeast and mammals begin to arise after the above two similar steps. Dihydrosphingosine is generally hydroxylated by the enzyme suppressor of rsv161/syringomycin response protein 2 (Sur2p/Syr2p), in* S. cerevisiae* to generate phytosphingosine. Ceramide synthases, Lag1 and Lac1, later acylate phytosphingosine while in mammalian cells dihydrosphingosine is straightforwardly acylated into dihydroceramide by the action of enzymes Lac1p and Lag1p. Longevity assurance gene cognate 1 (LAC1) and longevity assurance gene 1 (LAG1) encode the protein Lac1p and Lag1p, respectively. The yeast enzymes Lac1p and Lag1p have high homology to mammalian six Lass's (longevity assurance)/CerS1-6 [23]. CerS1-6 are known as (dihydro)ceramide synthases in mammals and translated by six different genes (*CERS1-6/LASS1-6*) that mainly localized into the ER. In mammals, dihydroceramide desaturase enzyme converts dihydroceramide into ceramide. In yeast, the following complex sphingolipids are synthesized from phytoceramide such as mannosylinositol phosphorylceramide (MIPC), inositol phosphorylceramide (IPC), and mannosyldiinositol phosphorylceramide (M(IP)_2_C). In case of mammals many complex sphingolipids, such as sphingomyelin, galactosylceramide, and glucosylceramide, are synthesized. Sphingosine is generated by the removal of an acetyl group from the ceramide through the action of many ceramidases. Ceramide phosphate is produced by the phosphorylation of ceramide via the sphingolipid pathway enzyme ceramide kinase which transfers phosphate group to ceramide [7, 23].

## 3. Structure and Function of Inositol Phosphosphingolipid Phospholipase C Proteins

Sawai et al. reported that gene* YER019w* decodes the IPS-PLC and possesses homology to bacterial neutral sphingomyelinase [24]. They named this gene as inositol phosphosphingolipid phospholipase C1 for IPS-PLC, while in mammals it has two homologs nSMase 1 and nSMase 2. N-SMase activity was first observed in patients with Niemann-Pick disease, who exhibit deficiencies in aSMase [25]. They also detected that upregulation of inositol phosphosphingolipid phospholipase C1 gene in* S. cerevisiae* dramatically increased N-SMase as well activities of all IPSs including (M(IP)_2_C), IPC, and MIPC and has a size of ~55 kDa [24]. Green fluorescent protein tagging analysis indicates that inositol phosphosphingolipid phospholipase C1 protein mainly localized to endoplasmic reticulum and mitochondria. Moreover, various groups have also shown that deletion of the inositol phosphosphingolipid phospholipase C gene causes absolute failure of phospholipase C (PLC) activity for all IPSs. This clearly suggests that inositol phosphosphingolipid phospholipase C is possibly the only known gene encoding IPS-PLC activities. The yeast N-SMases inositol phosphosphingolipid phospholipase C and CSS1 have nearly about 35% similarity to each other. Mammalian SMases nSMase 1 and nSMase 2 show about 28% homology to the yeast inositol phosphosphingolipid phospholipase C. Inositol phosphosphingolipid phospholipase C protein has two domains: the N-terminal catalytic active domain which presents outside the membrane and interact with the lipid substrates [24]. The second domain is known as C-terminal domain and shows interaction with the anionic phospholipids phosphatidylglycerol (PG), cardiolipin (CL), and phosphatidylserine (PS). Inositol phosphosphingolipid phospholipase C protein is generally activated by the cardiolipin (CL), phosphatidylglycerol (PG), and phosphatidylserine (PS). Mutation in three known extremely conserved amino acids, for example, E100, D233, and H334, of the catalytic domain of inositol phosphosphingolipid phospholipase C protein completely decreases the inositol phosphosphingolipid phospholipase C activity. The protein blast analysis of inositol phosphosphingolipid phospholipase C1 with bacterial sphingomyelinase indicates that residue E100 is likely to function in metal ion binding, the D234 residue works in an interaction with the phosphate group of sphingomyelin, and H344 is responsible for catalytic activity [26]. Mutation at site of D163 and K168 to alanine in the P-loop-like domain of inositol phosphosphingolipid phospholipase C1 completely abrogated inositol phosphosphingolipid phospholipase C protein activity, suggesting an essential role in catalysis for both amino acids. The amino acids D163 and K168 are conserved among all sphingomyelinases from bacteria to mammals [26]. In* Cryptococcus neoformans* IPC-PLC activity completely lost in case D114A and K119A mutations suggesting that these two amino acids are very crucial for the catalytic activity of* Cryptococcus neoformans* inositol phosphosphingolipid phospholipase C1 [6].

## 4. Role of Inositol Phosphosphingolipid Phospholipase C in DNA Damage Checkpoint

Many groups have already shown the relationship between ceramides and DNA damage response after the ionizing radiation [27]. The cross talk between ceramides and DNA damage is also well elucidated [28]. We and others have shown that inositol phosphosphingolipid phospholipase C1 plays a crucial part in determining the cellular morphology of the cells at the time of replication stress in yeast. Furthermore, we and others have also observed that inositol phosphosphingolipid phospholipase C is an important controller of cellular morphogenesis under various kinds of genotoxic and environmental stress [29]. During hydroxyurea induced replication stress cells lacking inositol phosphosphingolipid phospholipase C lead to morphological changes and aberration in actin depolymerization. It is well-known that hydroxyurea activates inositol phosphosphingolipid phospholipase C and increases the ceramides. The mediators proteins of replication checkpoint, topoisomerase I interacting factor (Tof1) and chromosome segregation in meiosis 3 (Csm3), do not play a foremost role in transduction of the signals generated in inositol phosphosphingolipid phospholipase C1 depleted cells during hydroxyurea treatment. However, inositol phosphosphingolipid phospholipase C functions in parallel with these checkpoint mediators and control the cell growth and morphology in normal cells. Rad9 the mediator of DNA damage induced checkpoint plays a crucial role to control signals generated at the time of replication stress in inositol phosphosphingolipid phospholipase C knockdown cells, leading to the cell morphological defects. It is reported by many groups that Rad53 controls cellular morphology in yeast cells. The DNA damage induced checkpoint effector protein Rad53 kinase, activated by Rad9 at the time of DNA damage, and also controls hydroxyurea dependent morphological abnormality in inositol phosphosphingolipid phospholipase C depleted cells. This clearly signifies that DNA damage induced checkpoint pathway molecules are active under these stress conditions and play an important role [30]. Interestingly, mitosis inhibitor protein kinase* SWE1* and cyclin-dependent kinase 1 (Cdk1) were found to control not only the cell cycle of yeast cells but also the morphological defects of inositol phosphosphingolipid phospholipase C depleted cells [31]. Lastly, it has been also shown that role of inositol phosphosphingolipid phospholipase C in the maintenance of cellular morphology was not restricted to only replication stress. This sphingolipid pathway gene was also well-known to regulate the shape of the cell and its morphology under other stress conditions, such as after treatment of galactose sugar and butanol alcohol [30, 32, 33].

## 5. Role of Inositol Phosphosphingolipid Phospholipase C in Pathogenesis

Inositol phosphosphingolipid phospholipase C one side plays a crucial role during the checkpoint while other ways have also shown that sphingolipid metabolism is closely linked to the virulence of pathogenesis of* Cryptococcus neoformans* (*Cn*). The* Cryptococcus neoformans* is a serious menace to immunocompromised, especially for the person suffering from the disease HIV and immunocompetent individuals also [12]. As we discussed earlier Inositol phosphosphingolipid phospholipase C has been characterized in* S. cerevisiae* [24]* Cryptococcus* [6] and* Leishmania* [34], suggesting that this sphingolipid pathway enzyme has the unique role in these organisms. Various groups have also shown by sphingolipidomic analysis that several phytoceramide subspecies increased in wild type cells after hydroxyurea treatment. The deletion of the inositol phosphosphingolipid phospholipase C gene in* S. cerevisiae* makes it more sensitive to hydroxyurea and methyl methanesulfonate and causes arrest of cell cycle and leads to morphological defects in cell shapes. In* Cryptococcus neoformans* inositol phosphosphingolipid phospholipase C plays a crucial role in hydroxyurea and methyl methanesulfonate tolerance. Hydroxyurea also affect the cell division in inositol phosphosphingolipid phospholipase C knockdown cells as compared to wild type cells, this leads to morphological changes (formation of cell chains and lawns kind of morphology) in inositol phosphosphingolipid phospholipase C depleted cells. Hydroxyurea also affects cell wall synthesis in these pathogens and actin polymerization during* Cryptococcus neoformans* cell division which could be a potential drug for treatment of these cells. We also observed that hydroxyurea treatment inhibits the growth of inositol phosphosphingolipid phospholipase C delta cells in the mouse. This clearly indicates that hydroxyurea treatment with inositol phosphosphingolipid phospholipase C deletion has a synergistic effect and this increasing host survival occurs due to decreasing organ fungal load [32]. In the lung of the mouse, hydroxyurea treatment reduced the number of colony forming units of* Cryptococcus neoformans* wild type cells nearly about tenfold from the initial inoculums as compared to untreated mice. Amazingly on the other hand the number of colony forming units of* Cryptococcus neoformans* inositol phosphosphingolipid phospholipase C depleted cells decreased more than thousandfold in hydroxyurea drug treated mice as compared to untreated mice [32].

*Leishmania* parasites cause a spectrum of diseases very well-known as leishmaniasis and infect more than twelve million people around the world.* Leishmania* also possesses inositol phosphosphingolipid phospholipase C homolog that is known as inositol phosphosphingolipid phospholipase C-Like (*ISCL*). Zhang et al. described that* Leishmania* parasites like cryptococcus inositol phosphosphingolipid phospholipase C-Like possesses neutral SMase activity and plays an essential role in sphingolipid degradation and* Leishmania major* virulence. They also observed that in BALB/c mice, knockdown of inositol phosphosphingolipid phospholipase C-like completely abolished acute disease pathology. Inositol phosphosphingolipid phospholipase C-like is also necessary for the production of IPC but is not indispensable for the production of ethanolamine (EtN) [34].

## 6. Future Directions

It is well-known that after hydroxyurea drug treatment the level of inositol phosphosphingolipid phospholipase C increases into the cell but the exact mechanism behind this process is not uncovered yet [35]. It will also be interesting to check what happened to inositol phosphosphingolipid phospholipase C after MMS treatment and the biochemical and molecular mechanism behind this. Activation and regulation of its mammalian homolog N-SMase and its role in DNA damage response are also not well elucidated. Role of SMases in cancer and drug target could be another interesting area that is less explored. Another predominantly fascinating field in this area is the involvement of fungal sphingolipid pathways in cell signaling and virulence. The role of inositol phosphosphingolipid phospholipase C is not well elucidated in pathogen like* Cryptococcus* and* Leishmania*. It will also be interesting to know how these pathways play the crucial role during DNA replication and checkpoint response. Another interesting area will be to explore the role of its mammalian homolog in cell division and apoptosis in various cancer models where it plays a crucial role. Knockdown of inositol phosphosphingolipid phospholipase C in combination with other drugs could be a novel tool for the treatment of pathogen induced diseases.
